# Comparative shotgun proteomic analysis of *Clostridium acetobutylicum *from butanol fermentation using glucose and xylose

**DOI:** 10.1186/1477-5956-9-66

**Published:** 2011-10-18

**Authors:** Kumaran Sivagnanam, Vijaya GS Raghavan, Manesh Shah, Robert L Hettich, Nathan C Verberkmoes, Mark G Lefsrud

**Affiliations:** 1 Department of Bioresource Engineering, Macdonald Campus, McGill University, Quebec, Canada; 2 Oak Ridge National Laboratory, Chemical and Life Sciences Divisions, Oak Ridge, TN, USA

**Keywords:** Butanol, ABE fermentation, *Clostridium acetobutylicum*, shotgun proteomics, mass spectrometry

## Abstract

**Background:**

Butanol is a second generation biofuel produced by *Clostridium acetobutylicum *through acetone-butanol-ethanol (ABE) fermentation process. Shotgun proteomics provides a direct approach to study the whole proteome of an organism in depth. This paper focuses on shotgun proteomic profiling of *C. acetobutylicum *from ABE fermentation using glucose and xylose to understand the functional mechanisms of *C. acetobutylicum *proteins involved in butanol production.

**Results:**

We identified 894 different proteins in *C. acetobutylicum *from ABE fermentation process by two dimensional - liquid chromatography - tandem mass spectrometry (2D-LC-MS/MS) method. This includes 717 proteins from glucose and 826 proteins from the xylose substrate. A total of 649 proteins were found to be common and 22 significantly differentially expressed proteins were identified between glucose and xylose substrates.

**Conclusion:**

Our results demonstrate that flagellar proteins are highly up-regulated with glucose compared to xylose substrate during ABE fermentation. Chemotactic activity was also found to be lost with the xylose substrate due to the absence of CheW and CheV proteins. This is the first report on the shotgun proteomic analysis of *C. acetobutylicum *ATCC 824 in ABE fermentation between glucose and xylose substrate from a single time data point and the number of proteins identified here is more than any other study performed on this organism up to this report.

## Introduction

*Clostridium acetobutylicum *is a gram positive, spore forming, obligate anaerobic bacteria and is one of the few microorganisms capable of converting a wide variety of sugars into three main products acetone, butanol and ethanol (ABE) [1]. ABE fermentation process was the primary source of butanol for over 40 years until the mid-1950s and is one of the oldest large-scale industrial fermentations [2]. ABE fermentation could not compete with the chemical synthesis of ABE solvents from petroleum since the mid-1950s [3]. However, increased concern over depletion of fossil fuels has led to renewed research interest in producing solvents via microbial fermentation processes.

Lignocellulosic biomass is an abundant renewable resource that can be used for the production of alternative fuels [4]. It is advantageous to use lignocellulosic biomass such as rice straw, wheat straw, corn stover and agricultural residues for biofuel production as they have limited impact on food supplies [5]. Glucose is the most abundant sugar found in lignocellulosic biomass with xylose being the second most abundant sugar [6]. *C. acetobutylicum *is able to ferment several pentose and hexose sugars [7] but the rate of uptake of the hexoses exceeds that of the pentoses [8]. Moreover, good solvent yields are obtained from glucose substrate whereas significantly lower values are found with xylose substrate utilized ABE fermentation [9]. The concurrent use of both sugars is a desirable characteristic of ABE fermentation from an economic point of view [10], proposing a substantial scope for investigation in *C. acetobutylicum*.

Classical product improvement strategies have been carried out by genetic manipulation and metabolic engineering of *C. acetobutylicum *for increased solvent production during ABE fermentation [11-13]. The physical and genetic map of *C. acetobutylicum *ATCC 824 has been constructed [14] and its genome was sequenced, elucidating 3.94-Mb chromosome and 192-kb megaplasmid that contains the majority of genes responsible for solvent production [15]. Primary annotation of *C. acetobutylicum *ATCC 824 categorized 3848 protein coding genes that include 2886 genes with assigned roles, 346 genes without any assigned roles, 575 conserved hypothetical genes and 41 hypothetical genes according to the comprehensive microbial resource [http://cmr.jcvi.org/cgi-bin/CMR/CmrHomePage.cgi].

The potential for improving the ABE fermentation process lies in the ability to gain a more complete understanding of *C. acetobutylicum*. Proteomics is a powerful tool to study the cellular mechanisms at the protein level and to understand the potential functions predicted by genome and transcriptome approaches. In turn, the proteomic knowledge can be used as targets for genetic and metabolic engineering [16,17]. Advances in the development of mass spectrometry have led to the possibility of studying the proteome of an organism. The objective of this work was to study the complete proteome of *C. acetobutylicum *from a single data point during ABE fermentation using glucose and xylose substrates by mass spectrometry (MS) based shotgun proteomics approach which relies on the identification of all proteins in a lysed cell mixture without the need for gel based separation techniques. Furthermore, this work provides a high throughput technique to study the *C. acetobutylicum *proteome, in addition to a valuable dataset of *C. acetobutylicum *proteins, thus providing a better understanding of the functional mechanisms of butanol production from glucose and xylose substrate in the ABE fermentation process.

## Materials and methods

### Strain and fermentation development

*C. acetobutylicum *ATCC-824 was obtained from American Type Culture Collection (ATCC, Cedarlane Labs, Burlington, Ontario, Canada) and was cultured using reinforced clostridial medium (RCM) in an anaerobic chamber (Coy Laboratory Products Inc., Grass Lake, Michigan, US) at 37°C for 20-24 h. Shake flask fermentation of *C. acetobutylicum *was performed in 250 ml anaerobic flask containing 100 ml of media consisting of (g/L) yeast extract (5), ammonium acetate (2), sodium chloride (1), KH_2_PO_4 _(0.75), K_2_HPO_4 _(0.75), cysteine HCl.H_2_O (0.50), MgSO_4 _(0.2), MnSO_4_.H_2_O (0.01), FeSO_4_.7H_2_O (0.01) and glucose (30) [18]. Shake flask fermentation was also performed using xylose of 30 g/L with the same media composition except glucose. Before inoculation, the medium was autoclaved at 121°C for 15 min (Cysteine HCl.H_2_O was filter sterilized through 0.45 μm filter and added to the medium) and cooled to 35°C in anaerobic chamber. The cell suspension was incubated at 37°C with shaking at 120 rpm and the growth was monitored with OD_600 nm_. Samples of 10 ml were harvested at the late exponential phase from the start of the inoculation from each fermentation experiment for further proteomic analysis. All chemicals used in this study were supplied from Fisher (Fisher Scientific, Canada) and Sigma (Sigma-Aldrich, Canada), unless otherwise specified.

### Cell lysis & protein extraction

The microbial cell pellets (~100 mg wet mass) from fermentation broth were processed through single tube whole cell lysis and protein digestion. Briefly, the cell pellet was resuspended in 6 M guanidine/10 mM dithiothreitol (DTT) with 5 0 mM Tris/10 mM CaCl_2 _at pH 7.6 by vortexing every 10 min for the first hour and incubated at 37°C for 12 hrs to lyse cells and extrude proteins. The guanidine concentration was diluted with six-fold 50 mM ris buffer/10 mM CaCl_2 _and 5-10 μg sequencing grade trypsin (Promega, Madison, WI, USA) was added and incubated at 37°C for 12 hrs to digest proteins to peptides. A second aliquot of the same amount of sequencing grade trypsin was added and incubated at 37°C for another 6 hrs to ensure the digestion process. 1 M DTT was added to a final concentration of 20 mM and incubated for another hour with gentle rocking at 37°C. The complex peptide solution was centrifuged at 10,000 g for 10 min to remove cellular debris and the supernatant was collected, cleaned using Sep-Pak plus (Waters Limited, Mississauga, Ontario, Canada) and concentrated. For each LC-MS/MS analysis below, ~1/4 of the total sample was used based on the protocol followed by Verberkmoes et al [19].

### Mass Spectrometry

Samples were analyzed in technical duplicates through a 2D nano-LC MS/MS system with a split-phase column (~3-5 cm SCX and 3-5 cm C18) (Polymicro technologies, Phoenix, AZ) [20] on a LTQ (ThermoFisher Scientific, San Jose, CA, USA) with 22 h runs [21,22]. The LTQ settings were as follows: all data-dependent MS/MS in LTQ (top five), two microscans for both full and MS/MS scans, centroid data for all scans and two microscans averaged for each spectrum, dynamic exclusion set at 1.

### Proteome informatics

All MS/MS spectra were searched with the SEQUEST algorithm [23] against a *C. acetobutylicum *Uniprot proteome databases [24] and filtered with DTASelect/Contrast [20] at the peptide level (Xcorrs of at least 1.8 [+1], 2.5 [+2], 3.5 [+3]). Only proteins identified with two fully tryptic peptides from a 22 h run were considered for further biological study. An in-house script was used to extract protein identifications, peptides, spectra, and sequence coverage from DTASelect filtered output files and used in calculation of protein abundance determination.

### False positive rate

The overall false positive rate (FPR) was estimated by doubling the number of peptides found from the reverse database and dividing the result by the total number of identified peptides from both databases using the formula: % fal = 2[n_rev_/(n_rev _+ n_real_)]*100 where % fal is the estimated false positive rate, n_rev _is the number of peptides identified from the reverse database and n_real _is the number of peptides identified from the real database [25].

### Relative protein abundance

The relative abundances of thousands of proteins identified during MS analysis were estimated by calculating the normalized spectral abundance factors (NSAF). The NSAF for a protein is the number of spectral counts (SpC, the total number of MS/MS spectra) identifying a protein, divided by the protein's length (L), divided by the sum of SpC/L for all proteins in the experiment [26,27].

## Results

### Shotgun proteomics of *C. acetobutylicum *from ABE fermentation

ABE fermentation of *C. acetobutylicum *ATCC 824 using glucose and xylose substrate were examined. Growth profiles of the two substrates were recorded by measuring the optical density (OD) of biomass at 600 nm and plotted against time (Additional file 1). Glucose was found to be preferred carbon source for *C. acetobutylicum *with the total biomass concentration reaching the peak OD_600 _of 1.76 in 30 h when compared to the xylose substrate with the total biomass concentration reaching the peak OD of 1.61 in 42 h. This demonstrated that *C. acetobutylicum *ATCC 824 could not utilize xylose substrate as efficient as glucose substrate utilized ABE fermentation process. In general, ABE fermentation undergoes acidogenesis in the early exponential phase and a major metabolic shift takes place which then switches to solventogenesis at the end of the exponential growth phase [28]. Proteome analysis of *C. acetobutylicum *was carried out from the samples collected at the late exponential phases of glucose and xylose utilized ABE fermentation.

**Figure 1:**
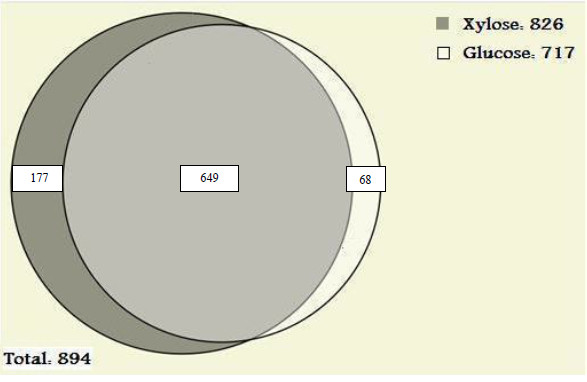
Venn diagram of the proteins identified in *C. acetobutylicum *between glucose and xylose utilized ABE fermentation

Our results present the first large scale investigation of the *C. acetobutylicum *proteome from a single time data point during ABE fermentation process using either glucose or xylose substrates by shotgun proteomics approach. The shotgun approach used enabled us to detect proteins by matching peptide mass data to available genome sequence databases. All proteins in the non-redundant Uniprot proteome database [http://www.uniprot.org] using keyword "*C. acetobutylicum*" that could match with the same set of peptides were included in the protein list. The total number of proteins identified from searching the database were 894 non redundant proteins, with 750 - 950 proteins identified per sample and replicate (Table 1). A total of 717 proteins and 826 proteins were identified from the ABE fermentation using either glucose and xylose substrates respectively and 649 proteins were found to be commonly present in both the substrates (Figure 1). The false positive rate was calculated as 4.38% and 2.84% for the first and second MS runs respectively for the ABE fermentation from the glucose substrate and 3.84% and 1.26% for the first and second MS run respectively for the ABE fermentation from the xylose substrate.

**Table 1:** Number of protein, peptide and spectra identifications for proteins identified from ABE fermentation using different substrates (two technical runs each)

Sample ID	Protein identification	Peptide identifications	MS/MS spectra
**Glucose substrate**			

Run 1	801	7851	40064

Run 2	759	7565	43931

**Xylose substrate**			

Run 1	866	6746	32213

Run 2	939	8408	38934

### Label-free estimation of relative protein abundance

**Figure 2:**
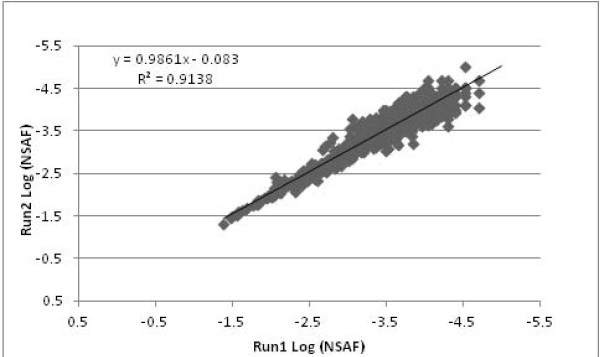
**Comparison of NSAF values**. ABE fermentation using glucose substrate, run 1 and run 2 NSAF values are plotted on a log scale. The solid squares represent the individual proteins identified in the MS runs

**Figure 3:**
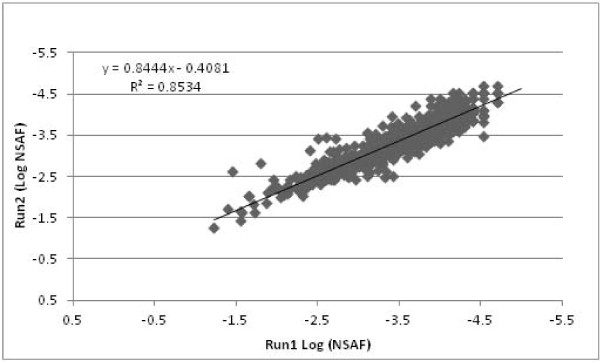
**Comparison of NSAF values**. ABE fermentation using xylose substrate, run 1 and run 2 NSAF values are plotted on a log scale. The solid squares represent individual proteins identified in the MS runs

**Figure 4:**
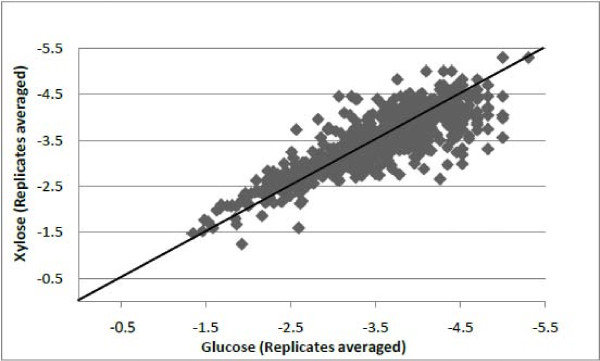
**Comparison of NSAF values for glucose and xylose substrates**. NSAF values were averaged amongst two individual technical runs per sample and plotted on a log scale. The solid squares represent the proteins identified in each sample. The straight diagonal line is for visualizing the location of all proteins that had approximately equal expression in both samples

The entire lists of proteins were sorted by averaged NSAF across both samples from the glucose and xylose substrates and the technical runs (Additional file 1). Comparing the NSAF data from each sample and technical run resulted in highly reproducible data; R^2 ^values of 0.91 (Figure 2) and 0.85 (Figure 3) for ABE fermentation samples using glucose and xylose respectively. The NSAF values for ABE samples using glucose and xylose substrates were averaged among their individual technical runs and compared to determine the unique and shared proteins (Figure 4). Based on the prediction of NSAF values, five most abundant proteins were found to be present in *C. acetobutylicum *from both glucose and xylose utilized ABE fermentation process. These include a heat shock protein, 60 kDa chaperonin, glyceraldehyde-3-phosphate dehydrogenase, phophocarrier protein and acetyl-CoA acetyl transferase. However, the remaining top five proteins for the glucose substrate were aldehyde-alcohol dehydrogenase, chaperone protein dnaK, 50S ribosomal protein L7/L12, fructose bisphophate aldolase, and electron transfer flavoprotein, while the remaining top five proteins for the xylose substrate were a cold shock protein, rare lipoprotein A, 10 kDa chaperonin, and two rubrerythrin proteins.

### Functional categorization of identified *C. acetobutylicum *proteins

A total of 657 proteins out of the 894 proteins identified in the analysis were assigned to 82 pathways which can be classified into 18 categories involved in *C. acetobutylicum *based on the KEGG annotation database. These include 568 proteins that were found to be commonly present in both substrates, 34 proteins identified only in glucose and 55 proteins only in xylose (Table 2). The proteins identified assigned to various pathways include carbohydrate metabolism (169), amino acid metabolism (105), translation (75), nucleotide metabolism (56), lipid metabolism (26), membrane transport (28), energy metabolism (21), metabolism of cofactors and vitamins (35), replication and repair (24), cell motility (17).

**Table 2:** Proteins identified in various pathways of *C. acetobutylicum *from glucose and xylose utilized ABE fermentation

Pathway classification	Glucose/Xylose	Glucose	Xylose
**Carbohydrate metabolism**	**138**	**07**	**24**

Glycolysis/Gluconeogenesis	18	-	03

Citrate cycle (TCA cycle)	07	-	-

Pentose phosphate pathway	11	01	03

Pentose and glucuronate interconversions	02	01	03

Fructose and mannose metabolism	14	01	01

Galactose metabolism	05	-	03

Starch and sucrose metabolism	10	-	06

Aminosugar and nucleotide sugar metabolism	21	02	05

Pyruvate metabolism	17	-	-

Glyoxylate and dicarboxylate metabolism	05	02	-

Propanoate metabolism	11	-	-

Butanoate metabolism	14	-	-

C5-Branched dibasic acid metabolism	02	-	-

Inositol phosphate metabolism	01	-	-

**Energy Metabolism**	**21**	**01**	**07**

Oxidative phosphorylation	07	-	-

Methane metabolism	09	01	03

Nitrogen metabolism	04	-	04

Sulfur metabolism	01	-	-

**Lipid Metabolism**	**26**	**00**	**02**

Fatty acid biosynthesis	10	-	01

Fatty acid metabolism	05	-	-

Synthesis and degradation of ketone bodies	03	-	-

Glycerolipid metabolism	03	-	-

Glycerophospholipid metabolism	03	-	-

Arachidonic acid metabolism	01	-	-

Biosynthesis of unsaturated fatty acids	01	-	01

**Nucleotide Metabolism**	**51**	**02**	**03**

Purine metabolism	32	-	02

Pyrimidine metabolism	19	02	01

**Amino Acid Metabolism**	**92**	**04**	**09**

Alanine, aspartate and glutamate metabolism	12	-	03

Glycine, serine and threonine metabolism	07	01	02

Cysteine and methionine metabolism	14	-	-

Valine, leucine and isoleucine degradation	06	-	-

Valine, leucine and isoleucine biosynthesis	11	-	-

Lysine biosynthesis	11	-	-

Lysine degradation	04	-	-

Arginine and proline metabolism	06	02	02

Histidine metabolism	01	-	01

Tyrosine metabolism	05	-	-

Phenylalanine metabolism	05	-	-

Tryptophan metabolism	03	-	-

Phenylalanine, tyrosine and tryptophan biosynthesis	07	01	01

**Metabolism of Other Amino Acids**	**19**	**00**	**00**

beta-Alanine metabolism	01	-	-

Taurine and hypotaurine metabolism	02	-	-

Selenocompound metabolism	04	-	-

Cyanoamino acid metabolism	04	-	-

D-Glutamine and D-glutamate metabolism	03	-	-

D-Arginine and D-ornithine metabolism	01	-	-

D-Alanine metabolism	02	-	-

Glutathione metabolism	02	-	-

**Glycan Biosynthesis and Metabolism**	**09**	**01**	**01**

Peptidoglycan biosynthesis	09	01	01

**Biosynthesis of Polyketides and Nonribosomal Peptides**	**03**	**01**	**00**

Polyketide sugar unit biosynthesis	03	01	-

**Metabolism of Cofactors and Vitamins**	**29**	**03**	**03**

Thiamine metabolism	04	01	-

Riboflavin metabolism	02	-	-

Vitamin B6 metabolism	02	-	-

Nicotinate and nicotinamide metabolism	03	-	01

Pantothenate and CoA biosynthesis	09	-	01

Folate biosynthesis	-	01	01

One carbon pool by folate	05	-	-

Porphyrin and chlorophyll metabolism	04	01	-

**Biosynthesis of Secondary Metabolites**	**12**	**02**	**00**

Terpenoid biosynthesis	05	-	-

Streptomycin biosynthesis	04	01	-

Novobiocin biosynthesis	03	01	-

**Xenobiotics Biodegradation and Metabolism**	**12**	**00**	**01**

Naphthalene degradation	02	-	-

Nitrotoluene degradation	01	-	01

Benzoate degradation	05	-	-

Chloroalkane and chloroalkene degradation	04	-	-

**Transcription**	**03**	**00**	**00**

RNA polymerase	03	-	-

**Translation**	**73**	**01**	**01**

Ribosome	45	-	01

Aminoacyl-tRNA biosynthesis	28	01	-

**Folding, Sorting and Degradation**	**15**	**01**	**00**

Protein export	04	-	-

Sulfur relay system	02	01	-

RNA degradation	09	-	-

**Replication and Repair**	**22**	**02**	**00**

DNA replication	04	01	-

Base excision repair	03	-	-

Nucleotide excision repair	04	01	-

Mismatch repair	06	-	-

Homologous recombination	05	-	-

**Membrane Transport**	**23**	**02**	**03**

ABC transporters	14	01	03

Phosphotransferase system (PTS)	06	01	-

Bacterial secretion system	03	-	-

**Signal Transduction**	**13**	**00**	**01**

Two-component system	12	-	01

**Cell Motility**	**10**	**07**	**00**

Bacterial chemotaxis	07	03	-

Flagellar assembly	03	04	-

**Total**	**568**	**34**	**55**

Our results demonstrate that the majority of the proteins involved in various *C. acetobutylicum *metabolic pathways were found to be commonly present with both glucose and xylose substrates. All the enzymes involved in the acid and solvent formation pathways were identified from both glucose and xylose substrates except alpha acetolactate decarboxylase. This includes pyruvate-formate lyase, acetolactate synthase, acetyl-CoA acetyltransferase, 3-hydroxybutyryl-CoA dehydrogenase, 3-hydroxybutyryl-CoA dehydratase, butyryl-CoA dehydrogenase, bifunctional acetaldehyde- CoA/alcohol dehydrogenase, NADH-dependent butanol dehydrogenase A & B, alcohol dehydrogenase, phosphate butyryltransferase, butyrate kinase, butyrate-acetoacetate CoA-transferase, acetoacetate decarboxylase, phosphotransacetylase and acetate kinase. However, proteins involved in specific processes such as carbohydrate metabolism and cell motility were found to be highly variable between the glucose and xylose utilized ABE fermentation process.

The major mechanism for carbohydrate uptake in *C. acetobutylicum *is the phosphotransferase (PTS) system [29] along with non-PTS transport systems which include ATP - driven transporters and other non-PTS permeases [30]. The PTS system which consists of a multiprotein complex that includes phosphocarrier protein (Hpr), phosphoenolpyruvate protein kinase (PTS system enzyme I), PTS enzyme II ABC component were identified in both glucose and xylose substrates. In addition, 14 proteins identified from both substrates were found to be involved in ABC transport system. *C. acetobutylicum *degrades various carbohydrates by converting them into an intermediate of one of the central carbohydrate-degrading pathways: glycolysis, pentose phosphate pathway [31,32]. Almost all the enzymes that are involved in glycolysis and pentose phosphate pathways were indentified in both glucose and xylose substrates. This includes glucokinase, glucose-6-phosphate isomerase, phosphofructokinase, fructosebisphosphate aldolase, triosephosphate isomerase, glyceraldehyde-3-phosphate dehydrogenase, phosphoglycerate kinase, enolase and pyruvate kinase. However, certain enzymes such as aldose-1-epimerase, fructose bisphosphate aldolase, xylulose kinase, arabinose isomerase were identified only in xylose and not in glucose, as they were not induced when glucose was used as substrate. Furthermore, enzymes involved in galactose, starch and sucrose metabolism identified in xylose were not found to be present in glucose utilized ABE fermentation. This could be attributed to a higher number of proteins identified in xylose than glucose utilized ABE fermentation process.

Chemotaxis proteins and flagellar assembly proteins are responsible for cellular motility and are mediated by motility related gene clusters [33,34]. Chemotaxis proteins indentified from both substrates include methyl accepting chemotaxis protein (MCP), chemotaxis histidine kinase - CheA, methylesterase - CheB/methylase - CheR, chemotaxis protein - CheC, chemotaxis signal receiving protein, chemotaxis response regulator - CheY. However, chemotaxis protein - CheV and chemotaxis signal transduction protein CheW were identified only in glucose and not in xylose substrate. The chemotaxis system senses the changes in pH, temperature, nutrient, toxin concentration, etc. and methyl accepting chemotaxis protein relay the detected environmental signals to the histidine kinase-CheA protein through the CheW coupling protein causing autophosphorylation (CheA-P). This phosphoryl group from CheA-P is transferred to response regulator- CheY that interacts with flagellar motor protein FliY. Besides, CheA-P can transfer phosphate to the response regulator-CheB that removes methyl groups from specific glutamate residues of MCP. The chemotaxis protein-CheC is involved in the coordination of the MCPs methylation process.

The FliY flagellar motor protein causes a change in the rotational direction of the flagellum resulting in swimming of the bacterium [35]. Regarding flagellar proteins, hook associated protein (flagellin family) - hag, flagellar hook associated protein - FliD, flagellar motor switch protein - FliY were identified from both substrates, whereas four proteins namely, flagellin, flagellin family protein, flagellar hook protein - FlgE, flagellar hook associated protein - FlgK were identified only in glucose and missing in the xylose substrate.

### Differentially expressed proteins

**Figure 5:**
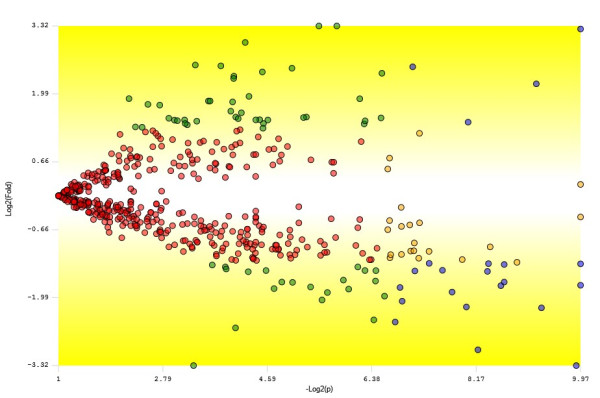
**TFold pairwise analysis of proteins identified from ABE fermentation using glucose and xylose substrates**. Each protein is represented as a dot and is mapped according to its log2 (fold change) on the ordinate axis and its -log2 (t-test p-value) on the abscissa axis. Refer to the text for the differentially expressed protein details

**Figure 6:**
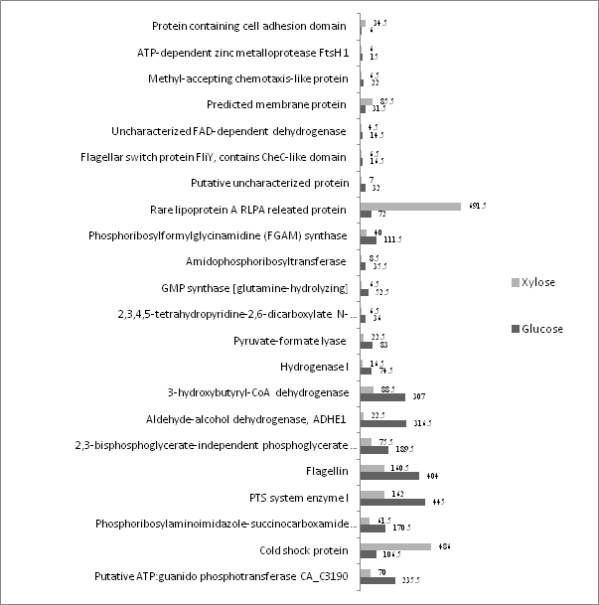
**Differentially expressed proteins identified from ABE fermentation between glucose and xylose substrates based on the spectral counting method with the spectral score shown at the end of each bar**.

Proteins that were identified from *C. acetobutylicum *during ABE fermentation from glucose and xylose substrates were examined for their differential expression using PatternLab software [36]. A TFold pairwise analysis of proteins identified from ABE fermentation using two different substrates were performed to pinpoint the differentially expressed proteins based on the spectral counting method (Figure 5). A total of 22 proteins (blue-dots) were found to be differentially expressed with an absolute fold change > 2.5 which is the established fold change cut-off and p-values < 0.05 considered as statistically significant. Out of these 22 significantly differentially expressed proteins, the expression levels of 18 proteins were found to be higher from glucose substrates and 4 proteins were from xylose substrate (Figure 6). Proteins such as cyclopropane fatty acid synthase, 50S ribosomal protein L17, signal peptidase I, queuosine biosynthesis protein, tRNA uridine 5-carboxymethylaminomethyl modification protein did not meet the fold-change cut-off but were indicated as statistically different (green-dots). Moreover, 12 proteins (orange-dots) met the fold-change cut-off but cannot be claimed to be statistically different and about 407 proteins (red-dots) did not satisfy the fold-change or the statistical cut-offs.

Aldehyde-alcohol dehydrogenase enzyme and 3-hydroxybutyryl-CoA dehydrogenase enzymes that were directly involved in the butanoate pathway were found to be highly up-regulated in glucose utilized ABE fermentation than xylose. The bi-functional aldehyde-alcohol dehydrogenase (AAD) protein is involved in the catalysis of the two step conversion of butyryl-CoA to butanol or of acetyl-CoA to ethanol [13]. 3-hydroxybutyryl-CoA dehydrogenase (HBD) enzyme in the central fermentation pathway is vital for the production of both acid and solvent. HBD catalyzes the reduction of acetoacetyl-CoA by NAD(P)H which is an initial and important process for the ultimate production of butyrate and butanol [37]. Similarly, pyruvate formate lyase (PFL) enzyme involved in the butanoate metabolism which converts pyruvic acid to formic acid [38] was also found to be upregulated in glucose than xylose substrate.

Proteins related to cellular motility were found to be up-regulated in glucose utilized ABE fermentation than xylose. These include, flagellin family - possible hook associated protein (FlaC), flagellar switch protein - contains CheC like domain (FliY) and methyl accepting chemotaxis like protein. The FlaC protein is one of the four proteins involved in flagellin structure that have been identified within a flagellar locus [39]. FliY is a flagellar motor switch protein responsible for the swimming of the bacterium by causing a change in the rotational direction of the flagellum. Methyl accepting chemotaxis proteins (MCPs) are transmembrane receptors that functions as a chemotaxis sensory transducer, transmitting the signal from the binding protein to the two - component system consisting histidine kinase (CheA) and the response regulator (CheY) [35].

Interestingly, rare lipoprotein A (RLPA) was found to be up-regulated in xylose but not the glucose substrate. Most lipoprotein act as membrane chaperones preventing unproductive interactions with the cell wall [40]. In addition, cold shock protein, protein containing cell adhesion domain and a predicted membrane protein were also found to be up-regulated in xylose substrate but not in glucose. However, not surprisingly, phosphoenolpyruvate-protein kinase (PTS system enzyme I) which is a membrane associated protein [41] was found to be up-regulated in glucose but not xylose. This observation is not unexpected as the specificity of *C. acetobutylicum *phosphotransferase (PTS) system varies for different sugars and glucose is preferred when compared to xylose [42]. A set of proteins which includes phosphoribosylaminoimidazole-succinocarboxamide synthase, GMP synthase, amidophosphoribosyltransferase, phosphoribosylformylglycinamidine (FGAM) synthase involved in purine metabolism were found to be up-regulated in glucose substrate. In addition, a putative uncharacterized protein (Q97LK2), 2,3-bisphosphoglycerate-independent phosphoglycerate mutase involved in biosynthesis of secondary metabolites, 2,3,4,5-tetrahydropyridine-2,6-dicarboxylate N-acetyltransferase involved in microbial metabolism and other proteins listed in Figure 6 were also found to be up-regulated in glucose.

## Discussion

A number of studies have been performed in *C. acetobutylicum *from ABE fermentation in order to achieve a better understanding of the butanol production process and limited proteome data are also available that have attempted to identify the protein composition of *C. acetobutylicum*. However, all proteomic studies published so far focused on acidogenic and solventogenic proteins involved in the metabolic pathways and the protein identification techniques were based on one and/or two dimensional - gel electrophoresis-mass spectroscopy method, exploring restricted number of proteins [43-46]. The proteome reference map published on *C. acetobutylicum *DSM 1731 strain identified about 564 proteins that accounts for 14.7% of the predicted genome and 416 proteins were used to reconstruct its metabolic network [45]. Another proteomic view published on acidogenic and solventogenic steady-state cells of *C. acetobutylicum *in chemostat culture identified 383 proteins [47]. In contrast, whole proteome investigation of *C. acetobutylicum *during ABE fermentation process have not been analyzed yet, which constituted the motivation of this study. This study is the first report on the whole proteome analysis of *C. acetobutylicum *ATCC 824 during ABE fermentation using glucose and xylose substrates, identifying 894 proteins from a single time data point through MS-based shotgun proteomics approach without the need for gel-based separation or *de novo *sequencing techniques. This 894 proteins account for 23.2% of the predicted 3848 ORFs in the *C. acetobutylicum *genome that includes 168 uncharacterized proteins out of the 346 genes without any assigned roles and the number of proteins identified in this study is more than any other proteomic study published on *C. acetobutylicum *so far. This 23.2% coverage of *C. acetobutylicum *ATCC 824 proteome based on gel-free shotgun proteomics approach is higher than the proteome reference map of other organisms such as 21.3% for *Bifidobacterium longum *NCC2705 [48], 16.9% for *Corynebacterium jeikeium *K411 [49], 12.8% for *Neisseria meningitidis serogroup *A [50], 9.8% for *Deinococcus geothermalis *[51], 5.7% for *Bacillus anthracis *A16R [52] which used two dimensional gel electrophoresis method. The extensive range of proteins identified in this paper on *C. acetobutylicum *can be potentially used to study this organism in-depth at proteome level.

The number of proteins identified in this *C. acetobutylicum *ATCC 824 proteome analysis is more when compared to the previous proteome reference map constructed using *C. acetobutylicum *DSM 1731 strain [45]. Out of the 177 proteins predicted to be involved in carbohydrate metabolism, we have identified a total of 140 and 151 proteins in ATCC 824 strain from glucose and xylose utilized fermentation respectively, when compared to only 98 proteins identified from DSM 1731 strain. Similarly, the number of proteins identified in mechanisms such as nucleotide metabolism, lipid metabolism, energy metabolism, and replication and repair processes were higher in this study. This is the first study to identify most of the enzymes involved in the acid and solvent formation pathway of *C. acetobutylicum *ATCC 824 including the alcohol dehydrogenase enzyme which was not identified in the proteome map of DSM 1731 strain. Moreover, this study is also in accordance with the recently reported membrane proteome analysis of *C. acetobutylicum *DSM 1731 [46]. Proteins such as glyceraldehydes-3-phosphate dehydrogenase and chaperonin proteins were found to be the most abundant of all the proteins identified in both the studies.

The most noticeable proteins identified in this study between glucose and xylose substrate utilized ABE fermentation were the proteins involved in flagellar assembly and bacterial chemotaxis which confers the cellular motility mechanisms. Flagellin (CAC1634), flagellin family protein (CAC2167), flagellar hook protein FlgE (CAC2154) that connects the basal body to the filaments and flagellar hook associated protein FlgK (CAC2212) which forms the junction between the hook and the filament were identified only in *C. acetobutylicum *from glucose substrate. The swimming characteristics of flagellar systems is a strong survival advantage in order to move to a more favourable environment due to changes in the pH, temperature, nutrient, toxin concentration that are detected by chemotaxis systems [35]. Chemotaxis signalling systems are highly sensitive to chemical cues that allow bacteria to track favourable chemical gradients in their environment [33]. The two proteins, receptor kinase coupling protein - CheW (CAC2217) and CheV (CAC1233) which is a two domain chemotaxis coupling protein that consists of CheW-like portion (CheVw) plus a receiver (REC) domain [53] were identified only from glucose and not in xylose. CheV has been previously described as a *Bacillus subtilis *protein homologous to CheW protein [54] and studies on *B.subtilis *mutants lacking both CheW and CheV proteins found that the mutant strains were non-chemotactic (Che^-^) [55]. This suggests that the absence of CheV and CheW proteins in *C. acetobutylicum *grown on xylose lost the chemotactic features and is non-chemotactic when compared to glucose substrate. Therefore, *C. acetobutylicum *in glucose utilized ABE fermentation is able to adjust or adapt to a stimulus triggered by the chemotactic responses that sense the chemical cues such as butanol toxicity which is a major issue for the fermentative production of butanol [3]. These results correlate well with the previous work done by Gutierrez and Maddox explaining that the motility of *C. acetobutylicum *during fermentation is a chemotactic response [56].

Comparative proteomic analysis revealed that aldehyde-alcohol dehydrogenase enzyme (CAP0035), 3-hydroxybutyryl-CoA dehydrogenase enzyme (CAC2708), flagellar motor switch protein - FliY (CAC2215) which controls the swimming of the bacterium, and flagellin family hook associated protein (CAC2203) were highly up-regulated in *C. acetobutylicum *from glucose utilized ABE fermentation than xylose. In addition, the NSAF values of the enzymes acetoacetyl-coenzyme A: acetate/butyrate coenzyme A-transferase (CoA-transferase) and butyraldehyde dehydrogenase (BAD) were found to be relatively abundant only on the glucose substrate. These results are consistent with the literature which demonstrate that a highly motile inoculum results in higher solvent production and non-motility leads to no solvent due to the loss of CoA-transferase and BAD enzymes that are directly involved in solvent production [57,58]. Recent transcriptomic studies of *C. acetobutylicum *growing on mixtures of glucose and xylose also reported that genes for chemotaxis proteins and flagellin biosynthesis are activated through glucose [59]. Therefore, the mechanism that *C. acetobutylicum *uses for both glucose and xylose sugar resulting in a preference for glucose [60] could also be attributed to the high motility nature of *C. acetobutylicum *grown on glucose compared to less motility with xylose substrate. On the other hand, the expression of rare lipoprotein-A (CAP0058) was found to be highly up-regulated in xylose utilized ABE fermentation than glucose substrate. Studies on the transcriptional analysis of butanol stress and tolerance in *C. acetobutylicum *showed that butanol stress induced the expression of gene which codes for the rare lipoprotein-A [61]. This confirms that butanol stress is higher in *C. acetobutylicum *using xylose substrate and results in overeexpression of RLPA protein when compared to glucose substrate. Furthermore, the cold shock protein (CAC2990) was also found to be highly up-regulated in xylose compared to glucose substrate.

Overall, this study provides an efficient, high throughput and rapid technique to study the *C. acetobutylicum *proteome and this data serves as a base for future investigations to compare and understand the different substrate utilization and regulation of butanol production in ABE fermentation process at the proteome level. Moreover, we envision this dataset as a useful source for researchers interested in the *C. acetobutylicum *proteomic studies and differences between glucose and xylose utilized ABE fermentation at proteome level.

## Competing interests

The authors declare that they have no competing interests.

## Authors' contributions

KS carried out the fermentation, protein extraction and drafted the manuscript. RLH and NCV contributed in LC-MS/MS analysis and MS carried out the mass spectrometry data analysis. VR critically revised the manuscript for intellectual content. MGL was responsible for the conception and coordination of this study. All authors edited the manuscript and approved the final version.

## Supplementary Material

Additional file 1 ***C. acetobutylicum *proteins identified in this study**. Glucose_Run1 - First MS run of *C. acetobutylicum *from ABE fermentation using glucose. Glucose_Run2 - Second MS run of *C. acetobutylicum *from ABE fermentation using glucose. Xylose_Run1 - First MS run of *C. acetobutylicum *from ABE fermentation using xylose. Xylose_Run2 - Second MS run of *C. acetobutylicum *from ABE fermentation using xylose. NSAF_Glucose - NSAF values for protein identified from Glucose_Run1 and 2. NSAF_Xylose - NSAF values for protein identified from Xylose_Run1 and 2. Growth curve - Growth pattern of ABE fermentation between glucose and xylose substrates. Click here for file

## References

[B1] Hüsemann MHW Papoutsakis ET Enzymes limiting butanol and acetone formation in continuous and batch cultures of Clostridium acetobutylicum Applied Microbiology and Biotechnology 1989 31 435 444 10.1007/BF00270772

[B2] Rogers P Genetics and biochemistry of Clostridium relevant to development of fermentation processes Advances in Applied Microbiology 1986 31 1 60

[B3] Jones D Woods D Acetone-butanol fermentation revisited Microbiology and Molecular Biology Reviews 1986 50 484 10.1128/mr.50.4.484-524.1986 PMC373084 3540574

[B4] Himmel ME Ding SY Johnson DK Adney WS Nimlos MR Brady JW Foust TD Biomass recalcitrance: engineering plants and enzymes for biofuels production Science 2007 315 804 10.1126/science.1137016 17289988

[B5] Sticklen MB Plant genetic engineering for biofuel production: towards affordable cellulosic ethanol Nature Reviews Genetics 2008 9 433 443 10.1038/nrg2336 18487988

[B6] Yanase H Sato D Yamamoto K Matsuda S Yamamoto S Okamoto K Genetic engineering of Zymobacter palmae for production of ethanol from xylose Applied and environmental microbiology 2007 73 2592 10.1128/AEM.02302-06 17308178 PMC1855588

[B7] Compere A Griffith W Evaluation of substrates for butanol production Dev Ind Microbiol;(United States) 1979 20 509 51710863099

[B8] Ounine K Petitdemange H Raval G Gay R Acetone-butanol production from pentoses by Clostridium acetobutylicum Biotechnology Letters 1983 5 605 610 10.1007/BF00130841

[B9] Yu E Saddler J Enhanced acetone butanol fermentation by Clostridium acetobutylicum grown on d xylose in the presence of acetic or butyric acid FEMS Microbiology Letters 1983 18 103 107

[B10] Ezeji T Qureshi N Blaschek H Bioproduction of butanol from biomass: from genes to bioreactors Current Opinion in Biotechnology 2007 18 220 227 10.1016/j.copbio.2007.04.002 17462877

[B11] Green E Bennett G Inactivation of an aldehyde/alcohol dehydrogenase gene fromClostridium acetobutylicum ATCC 824 Applied biochemistry and biotechnology 1996 57 213 221 10.1007/BF02941702 8669898

[B12] Mermelstein L Papoutsakis E Petersen D Bennett G Metabolic engineering of Clostridium acetobutylicum ATCC 824 for increased solvent production by enhancement of acetone formation enzyme activities using a synthetic acetone operon Biotechnology and bioengineering 1993 42 1053 1060 10.1002/bit.260420906 18613233

[B13] Nair R Papoutsakis E Expression of plasmid-encoded aad in Clostridium acetobutylicum M5 restores vigorous butanol production Journal of Bacteriology 1994 176 5843 8083176 10.1128/jb.176.18.5843-5846.1994 PMC196790

[B14] Cornillot E Nair R Papoutsakis E Soucaille P The genes for butanol and acetone formation in Clostridium acetobutylicum ATCC 824 reside on a large plasmid whose loss leads to degeneration of the strain Journal of Bacteriology 1997 179 5442 9286999 10.1128/jb.179.17.5442-5447.1997 PMC179415

[B15] Nolling J Breton G Omelchenko M Makarova K Zeng Q Gibson R Lee H Dubois J Qiu D Hitti J Genome sequence and comparative analysis of the solvent-producing bacterium Clostridium acetobutylicum Journal of Bacteriology 2001 183 4823 10.1128/JB.183.16.4823-4838.2001 11466286 PMC99537

[B16] Anglade P Demey E Labas V Le Caer JP Chich JF Towards a proteomic map of Lactococcus lactis NCDO 763 Electrophoresis 2000 21 2546 2549 10.1002/1522-2683(20000701)21:12<2546::AID-ELPS2546>3.0.CO;2-J 10939470

[B17] Guillot A Gitton C Anglade P Mistou MY Proteomic analysis of Lactococcus lactis, a lactic acid bacterium Proteomics 2003 3 337 354 10.1002/pmic.200390047 12627387

[B18] Qureshi N Meagher M Huang J Hutkins R Acetone butanol ethanol (ABE) recovery by pervaporation using silicalite-silicone composite membrane from fed-batch reactor of Clostridium acetobutylicum Journal of Membrane Science 2001 187 93 102 10.1016/S0376-7388(00)00667-0

[B19] Verberkmoes N Russell A Shah M Godzik A Rosenquist M Halfvarson J Lefsrud M Apajalahti J Tysk C Hettich R Shotgun metaproteomics of the human distal gut microbiota The ISME journal 2008 3 179 189 18971961 10.1038/ismej.2008.108

[B20] Tabb D McDonald W Yates J DTASelect and Contrast: tools for assembling and comparing protein identifications from shotgun proteomics Journal of proteome research 2002 1 21 26 10.1021/pr015504q 12643522 PMC2811961

[B21] Lo I Denef V VerBerkmoes N Shah M Goltsman D DiBartolo G Tyson G Allen E Ram R Detter J Strain-resolved community proteomics reveals recombining genomes of acidophilic bacteria Nature 2007 446 537 541 10.1038/nature05624 17344860

[B22] Ram R VerBerkmoes N Thelen M Tyson G Baker B Blake R Shah M Hettich R Banfield J Community proteomics of a natural microbial biofilm Science 2005 308 1915 10.1126/science. 1109070 15879173

[B23] Eng J McCormack A Yates J An approach to correlate tandem mass spectral data of peptides with amino acid sequences in a protein database Journal of the American Society for Mass Spectrometry 1994 5 976 989 10.1016/1044-0305(94)80016-2 24226387

[B24] Apweiler R Bairoch A Wu C Barker W Boeckmann B Ferro S Gasteiger E Huang H Lopez R Magrane M UniProt: the universal protein knowledgebase Nucleic acids research 2004 32 D115 10.1093/nar/gkh131 14681372 PMC308865

[B25] Peng J Elias J Thoreen C Licklider L Gygi S Evaluation of multidimensional chromatography coupled with tandem mass spectrometry (LC/LC-MS/MS) for large-scale protein analysis: the yeast proteome Journal of proteome research 2003 2 43 50 10.1021/pr025556v 12643542

[B26] Florens L Carozza MJ Swanson SK Fournier M Coleman MK Workman JL Washburn MP Analyzing chromatin remodeling complexes using shotgun proteomics and normalized spectral abundance factors Methods 2006 40 303 311 10.1016/j.ymeth.2006.07.028 17101441 PMC1815300

[B27] Zybailov BL Florens L Washburn MP Quantitative shotgun proteomics using a protease with broad specificity and normalized spectral abundance factors Mol BioSyst 2007 3 354 360 10.1039/b701483j 17460794

[B28] Zheng YN Li LZ Xian M Ma YJ Yang JM Xu X He DZ Problems with the microbial production of butanol Journal of Industrial Microbiology and Biotechnology 2009 36 1127 1138 10.1007/s10295-009-0609-9 19562394

[B29] Mitchell WJ Tangney M Carbohydrate uptake by the phosphotransferase system and other mechanisms Handbook on Clostridia Boca Raton, Taylor & Francis 2005 155 175

[B30] Saier M Jr Fagan M Hoischen C Reizer J Transport mechanisms Bacillus subtilis and other gram-positive bacteria: biochemistry, physiology, and molecular genetics American Society for Microbiology, Washington, DC 1993 133 156

[B31] Steinmetz M Carbohydrate catabolism: pathways, enzymes, genetic regulation, and evolution Bacillus subtilis and other gram-positive bacteria: biochemistry, physiology, and molecular genetics American Society for Microbiology, Washington, DC 1993 157 170

[B32] Shimizu T Ohtani K Hirakawa H Ohshima K Yamashita A Shiba T Ogasawara N Hattori M Kuhara S Hayashi H Complete genome sequence of Clostridium perfringens, an anaerobic flesh-eater Proceedings of the National Academy of Sciences of the United States of America 2002 99 996 10.1073/pnas.022493799 11792842 PMC117419

[B33] Wadhams GH Armitage JP Making sense of it all: bacterial chemotaxis Nature Reviews Molecular Cell Biology 2004 5 1024 1037 10.1038/nrm1524 15573139

[B34] Paredes CJ Alsaker KV Papoutsakis ET A comparative genomic view of clostridial sporulation and physiology Nature Reviews Microbiology 2005 3 969 978 10.1038/nrmicro1288 16261177

[B35] Doß S Gröger C Knauber T Whitworth DE Treuner-Lange A 24 Comparative Genomic Analysis of Signal Transduction Proteins in Clostridia 2005

[B36] Carvalho P Fischer J Chen E Yates J Barbosa V PatternLab for proteomics: a tool for differential shotgun proteomics BMC bioinformatics 2008 9 316 10.1186/1471-2105-9-316 18644148 PMC2488363

[B37] Boynton ZL Bennet G Rudolph FB Cloning, sequencing, and expression of clustered genes encoding beta-hydroxybutyryl-coenzyme A (CoA) dehydrogenase, crotonase, and butyryl-CoA dehydrogenase from Clostridium acetobutylicum ATCC 824 Journal of Bacteriology 1996 178 3015 8655474 10.1128/jb.178.11.3015-3024.1996 PMC178046

[B38] Wang S Zhang Y Dong H Mao S Zhu Y Wang R Luan G Li Y Formic Acid Triggers the" Acid Crash" of Acetone-Butanol-Ethanol Fermentation by Clostridium acetobutylicum Applied and environmental microbiology 2011 77 1674 10.1128/AEM.01835-10 21216898 PMC3067271

[B39] Logan SM Flagellar glycosylation-a new component of the motility repertoire? Microbiology 2006 152 1249 10.1099/mic.0.28735-0 16622043

[B40] Wahlstrom E Vitikainen M Kontinen VP Sarvas M The extracytoplasmic folding factor PrsA is required for protein secretion only in the presence of the cell wall in Bacillus subtilis Microbiology 2003 149 569 10.1099/mic.0.25511-0 12634326

[B41] Desvaux M Khan A Scott-Tucker A Chaudhuri RR Pallen MJ Henderson IR Genomic analysis of the protein secretion systems in Clostridium acetobutylicum ATCC 824 Biochimica et Biophysica Acta (BBA)-Molecular Cell Research 2005 1745 223 253 10.1016/j.bbamcr.2005.04.006 15950297

[B42] Yu Y Tangney M Aass HC Mitchell WJ Analysis of the mechanism and regulation of lactose transport and metabolism in Clostridium acetobutylicum ATCC 824 Applied and environmental microbiology 2007 73 1842 10.1128/AEM.02082-06 17209069 PMC1828815

[B43] Schaffer S Isci N Zickner B Dürre P Changes in protein synthesis and identification of proteins specifically induced during solventogenesis in Clostridium acetobutylicum Electrophoresis 2002 23 110 10.1002/1522-2683(200201)23:1<110::AID-ELPS110>3.0.CO;2-G 11824611

[B44] Sullivan L Bennett GN Proteome analysis and comparison of Clostridium acetobutylicum ATCC 824 and Spo0A strain variants Journal of Industrial Microbiology and Biotechnology 2006 33 298 308 10.1007/s10295-005-0050-7 16308714

[B45] Mao S Luo Y Zhang T Li J Bao G Zhu Y Chen Z Zhang Y Li Y Ma Y Proteome reference map and comparative proteomic analysis between a wild type Clostridium acetobutylicum DSM 1731 and its mutant with enhanced butanol tolerance and butanol yield Journal of proteome research 2010 9 3046 3061 10.1021/pr9012078 20426490

[B46] Mao S Luo Y Bao G Zhang Y Li Y Ma Y Comparative analysis on the membrane proteome of Clostridium acetobutylicum wild type strain and its butanol-tolerant mutant Mol BioSyst 2011 7 1660 1667 10.1039/c0mb00330a 21384033

[B47] Janssen H Döring C Ehrenreich A Voigt B Hecker M Bahl H Fischer RJ A proteomic and transcriptional view of acidogenic and solventogenic steady-state cells of Clostridium acetobutylicum in a chemostat culture Applied Microbiology and Biotechnology 2010 1 18 10.1007/s00253-010-2741-x PMC3227527 20617312

[B48] Yuan J Zhu L Liu X Li T Zhang Y Ying T Wang B Wang J Dong H Feng E A proteome reference map and proteomic analysis of Bifidobacterium longum NCC2705 Molecular & Cellular Proteomics 2006 5 1105 10.1074/mcp.M500410-MCP200 16549425

[B49] Hansmeier N Chao TC Daschkey S Müsken M Kalinowski J Pühler A Tauch A A comprehensive proteome map of the lipid requiring nosocomial pathogen Corynebacterium jeikeium K411 Proteomics 2007 7 1076 1096 10.1002/pmic.200600833 17352426

[B50] Bernardini G Renzone G Comanducci M Mini R Arena S D'Ambrosio C Bambini S Trabalzini L Grandi G Martelli P Proteome analysis of Neisseria meningitidis serogroup A Proteomics 2004 4 2893 2926 10.1002/pmic.200400946 15378741

[B51] Liedert C Bernhardt J Albrecht D Voigt B Hecker M Salkinoja Salonen M Neubauer P Two dimensional proteome reference map for the radiation resistant bacterium Deinococcus geothermalis Proteomics 2010 10 555 563 10.1002/pmic.200800657 19941306

[B52] Wang J Ying T Wang H Shi Z Li M He K Feng E Yuan J Li T 2 D reference map of Bacillus anthracis vaccine strain A16R proteins Proteomics 2005 5 4488 4495 10.1002/pmic.200401322 16294314

[B53] Ottemann KM Alexander RP Lowenthal AC Harshey RM CheV: CheW-like coupling proteins at the core of the chemotaxis signaling network Trends in microbiology 2010 18 494 503 10.1016/j.tim.2010.07.004 20832320 PMC2975053

[B54] Fredrick KL Helmann JD Dual chemotaxis signaling pathways in Bacillus subtilis: a sigma D-dependent gene encodes a novel protein with both CheW and CheY homologous domains Journal of Bacteriology 1994 176 2727 8169223 10.1128/jb.176.9.2727-2735.1994 PMC205414

[B55] Rosario M Fredrick KL Ordal GW Helmann JD Chemotaxis in Bacillus subtilis requires either of two functionally redundant CheW homologs Journal of Bacteriology 1994 176 2736 8169224 10.1128/jb.176.9.2736-2739.1994 PMC205415

[B56] Gutierrez NA Maddox IS Role of chemotaxis in solvent production by Clostridium acetobutylicum Applied and environmental microbiology 1987 53 1924 16347417 10.1128/aem.53.8.1924-1927.1987 PMC204026

[B57] Petersen DJ Bennett GN Enzymatic characterization of a nonmotile, nonsolventogenicClostridium acetobutylicum ATCC 824 mutant Current Microbiology 1991 23 253 258 10.1007/BF02092026

[B58] Lyristis M Boynton ZL Petersen D Kan Z Bennett GN Rudolph FB Cloning, sequencing, and characterization of the gene encoding flagellin, flaC, and the post-translational modification of flagellin, FlaC, from Clostridium acetobutylicum ATCC824 Anaerobe 2000 6 69 79 10.1006/anae.1999.0311

[B59] Grimmler C Held C Liebl W Ehrenreich A Transcriptional analysis of catabolite repression in Clostridium acetobutylicum growing on mixtures of d-glucose and d-xylose Journal of biotechnology 2010 150 315 323 20883732 10.1016/j.jbiotec.2010.09.938

[B60] Ezeji T Blaschek HP Fermentation of dried distillers' grains and solubles (DDGS) hydrolysates to solvents and value-added products by solventogenic clostridia Bioresource technology 2008 99 5232 5242 10.1016/j.biortech.2007.09.032 17967532

[B61] Tomas CA Beamish J Papoutsakis ET Transcriptional analysis of butanol stress and tolerance in Clostridium acetobutylicum Journal of Bacteriology 2004 186 2006 10.1128/JB.186.7.2006-2018.2004 15028684 PMC374415

